# Facing the paradox of professionalizing peer roles in MH services: how addressing self-disclosure with self-determination theory might help

**DOI:** 10.1017/S2045796024000751

**Published:** 2025-01-13

**Authors:** Galia S. Moran

**Affiliations:** Department of Social Work, Ben-Gurion University, Beer Sheva, Israel

**Keywords:** system change, recovery orientation, peer roles, mental health services

## Abstract

Peer Support Workers (PSWs) play a crucial role in recovery-oriented mental health services. They offer support and hope by sharing their personal experiences and recovery journeys. However, transitioning from voluntary self-help roles to paid positions within statutory systems is not merely a technical shift. This change creates inherent tensions and conflicts, stemming from the integration of a peer model within a medical framework. I refer to the interface between these models as the “Professional-Peer Paradox” (PPP). At its heart, this paradox questions whether and how PSWs can integrate a role that relies on self-disclosure of shared lived experiences within a system rooted in professional knowledge norms delivered unidirectionally to service recipients. Using a whole organizational approach, I propose leveraging the autonomy-supportive environment concept from self-determination theory (SDT; Deci & Ryan, 2000) to promote self-disclosure in mental health services. I highlight the complexities involved in Peer Support Workers’ (PSWs) use of self-disclosure (lived experience) within statutory mental health (MH) services. I suggest that PSWs can better commit to their unique roles by structuring multiple peer roles with varying levels of self-disclosure and creating a culture that fosters peer practice. Overall, applying a SDT systems’ framework to the practice of self-disclosure can enhance the occupational identity of PSWs, establishing their unique position within the spectrum of mental health professions globally.

Peer support in mental health originated in non-governmental organizations and self-help groups. Over the past decades, peer support workers (PSWs) have increasingly been trained and employed via government- and insurance-based services (Farkas and Boevink, 2018; Shalaby and Agyapong, 2020; Slade *et al.*, 2014). They are now considered an essential element in the mental health (MH) field and indispensable in recovery-oriented care (Corrigan, 2024; Slade *et al.*, 2008, 2012; Stratford *et al.*, 2017).

Unlike conventional care, the peer model involves a non-hierarchical, reciprocal and person-centred approach. MH peer support has been defined as ‘offering and receiving help, based on shared understanding, respect and mutual empowerment between people in similar situations’ (Mead *et al.*, 2001). PSWs’ bring knowledge and skills that complement clinical and academic services in MH systems. The mechanisms of peer support involve sharing knowledge from experience, serving as role-models for the possibility of recovery by means of reciprocal empathic relationships in order to inspire hope and provide support (Farkas and Boevink, 2018).

Studies have demonstrated the far-reaching impact of peer support on personal recovery and quality of life indicators (Chinman *et al.*, 2015; Fortuna *et al.*, 2022). When all works well, benefits can be threefold: improved recovery outcomes for service users, enhancement of recovery orientation among MH staff-services and recovery gains for the peers themselves. For example, service users gain increased hope, self-esteem, empowerment and social inclusion as well as a decrease in hospitalization; PSWs can act as agents of change within MH services, by strengthening a recovery orientation and improving attitudes by staff towards service users; and PSWs themselves experience empowerment, enhanced social networks, better functioning, illness management, employability, skills and career development (Chinman *et al.*, 2015; Davidson *et al.*, 2012; Fortuna *et al.*, 2019; Moran *et al.*, 2012; Repper and Carter, 2011).

## The challenge of the professional–peer paradox and self-disclosure

Policy makers and directors of mental health services show increasing interest in hiring peer specialists to provide a range of services and supports to persons with severe mental illnesses (Chinman *et al.*, 2014; Davidson *et al.*, 2012; Mancini *et al.*, 2018; Mutschler *et al.*, 2022). As peer support progresses from a self-help model towards a standardized practice with an occupational identity, it inevitably faces challenges for its optimal integration in statutory MH services. Occupational peer support work-roles vary across MH services and can involve providing peer support in inpatient and outpatient settings, in individual or group formats, for specific targets (health related topics, personal medication, rights, etc.) or more generic befriending and support purposes, etc. (see also Chinman *et al.*, 2014; Fortuna *et al.*, 2022). Despite the diversity in PSW roles, they all share the commonality of being paid positions that provide peer support to mental health service users. To clarify, I am addressing these occupational, paid forms of peer support delivered and implemented within mental health systems, and not other forms of peer support in non-governmental organizations and grassroots initiatives. Henceforth, I will refer to them as PSWs and to the services they deliver as peer support services.

In contrast to working in a non-governmental organization/self-help framework, a PSW in a conventional/statutory mental health service often faces an unremitting challenge. This challenge involves staying true to the PSW role, which includes disclosing lived experience, mutuality, and being a role model for recovery. At the same time, PSWs must adhere to the norms of the traditional MH system within which they work. MH systems often minimize or discredit the value of sharing of lived experience and recovery orientation. This ongoing tension experienced by PSWs in MH services can be termed the professional–peer paradox. On one side of the paradox is the expectation for a peer relationship based on authentic self-disclosure, empathy, and eye-level camaraderie. On the other side is the requirement for formal, unidirectional expert-to-recipient service provision.

Thus, as a new practice, peer support services need to be integrated into the traditional methods by which community and clinical mental health organizations interact with, treat, and respond to their clients (Byrne *et al.*, 2022; Mancini, 2018; Moran, 2017). Mental health agencies struggle to effectively integrate and utilize PSWs. PSWs experience multiple challenges including include stigma, alienation, unclear work roles, skill deficits, lack of training opportunities, burnout and low payment (Adams, 2020; Ahmed *et al.*, 2015; Gates and Akabas, 2007; Mancini and Lawson, 2009; Moran *et al.*, 2013; Salzer *et al.*, 2010; Walker and Bryant, 2013). They run the danger of lacking important factors in job satisfaction such as role clarity, autonomy, respect and supervisor understanding of job role (Adams, 2020; Cronise *et al.*, 2016; Gagne *et al.*, 2018).

Numerous efforts have been made to address these challenges and facilitate the integration of peer support services into MH systems. These efforts include developing peer training programs that focus on peer values and skills, preparing organizational readiness, educating staff, defining clear role descriptions, providing supervision and fostering peer networking (Charles *et al.*, 2021; Chinman *et al.*, 2017; Corrigan, 2024; Cronise *et al.*, 2016; Moran, 2017; Mutschler *et al.*, 2022; Repper and Carter, 2011). Additionally, research agendas have been proposed for evaluating implementation, as well as for developing and testing professional development opportunities. (Byrne et al., 2022; Chinman *et al.*, 2017; Chinman *et al.*, 2017; Ibrahim *et al.*, 2020; Mancini, 2019; Moran, 2017; Moran *et al.*, 2020). Such investments have been fruitful to substantial degrees, especially in support of PSWs’ further occupational development and integrating the professional responsibilities required in MH services.

However, the inherent difference between the peer model (which lie at the core of peer support services) and the medical model put PSWs at continuous risk of being disempowered and misunderstood in their use of lived-experience in MH services. PSWs are liable to experience explicit and implicit pressures to become professionalized in the conventional sense (Byrne *et al.*, 2022). The demands and norms of the medical model might sap PSWs’ unique qualities and skills, thereby eroding the essence of peerness from their role (Corrigan, 2024). As a result, PSWs may often revert to traditional mental health worker roles, retreating from their unique peer support functions (Cronise *et al.*, 2016; Moran, 2017; Repper and Carter, 2011; Slade *et al.*, 2014).

Specifically, I would like to highlight the challenge of self-disclosure (i.e. use of lived experience) of PSWs in the context of mental health statutory services as core to the professional–peer paradox. PSWs are expected to leverage their personal lived experience to benefit others. Within this framework, **self-disclosure** serves as the central means for PSWs to transfer knowledge transfer to both service users and staff. The professional–peer paradox stems from the tension between PSWs’ use of self-disclosure (i.e. use of lived experience), which enhances mutual relationships and the norm in conventional MH settings, which are often characterized by formal and unidirectional consumer-provider relationships.

In MH settings, self-disclosure is considered a rare intervention that is to be employed with caution. Indeed in the past self-disclosure by therapists was taboo in traditional therapeutic settings. However, in recent years some schools of practice have come to recognize its value (Ben-Dor *et al.*, 2024; Hill *et al.*, 2018). Thus, integrating PSWs roles inherently disrupts the exisiting relational-dynamics and power structures within traditional MH care (also suggested by others too – e.g. Byrne *et al.*, 2022; Corrigan, 2024; Mancini, 2019).

More specifically, I believe it is self-disclosure of lived experience which lies at the crux of peer support model – a gift that other mental health practitioners cannot offer. Therefore, when implementing MH peer support, it is cruical to ask: How can we support PSWs in using their lived experience within traditional, medically modelled settings?

Self-disclosure is not an all-or-none practice in and of itself. Rather self-disclosure is a complex and dynamic act, which can change over time, develop with skill and vary with experience and context. Self-disclosure can be full or partial; differ according to the target of self-disclosure (e.g., service users, staff members or directors); and manifest differently according to one’s goals and situational factors (e.g. when providing crisis intervention, navigating the system, entering employment, rehab housing, etc.). Furthermore, practicing self-disclosure can depend on one’s own recovery processes, one’s sense of self, as well as self-stigma and lowered sense of status. Employing self-disclosure can also risk eliciting stigmatic attitudes and responses by service users and colleagues (Bril-Barniv *et al.*, 2017; Mancini, 2019; Moran *et al.*, 2013; Tomas *et al*., 2022). Thus, it is not surprizing that self-disclosure can be cognitively and emotionally taxing for PSWs (Mancini and Lawson, 2009).

Efforts to understand and support PSWs’ self-disclosure have been developed in recent years. For example, Mancini (2019) reported how peers engaged in a reflexive process to strategically use their personal illness and recovery stories to help others re-story their life narratives in addition to other communication strategies. Grundman, Edri *et al*., (2021), developed a coproduced training and working model for PSWs to support the practice of self-disclosure in MH settings. Their training focuses on three key elements: using components of lived-experience, structuring a personal ‘library’ of one’s accumulated knowledge from experience, and developing a guiding technique for retrieval of appropriate parts to share with service users of one’s lived-experience. Such research and training programmes can support the development of a discourse around self-disclosure and its complexity, allowing better grounding for PSWs to navigate their awareness and personal practice of self-disclosure.

The organizational level adds another layer of difficulty for the practice of self-disclosure – PSWs may be encouraged (or directed) to self-disclose according to ways that align with organizational preferences. Personal stories might be used in ways that are persuasive and potentially biasing towards organizational goals. For instance PSWs may be expected to persuade service users to align with clinicians’ preferences (Mancini, 2018; Mead *et al.*, 2013; Moran *et al.*, 2013; Tomas *et al*., 2022). In a recent qualitative study involving interviews with 29 mental health (MH) staff members and 13 PSWs across various MH agencies, it was found that staff and directors often tended to discourage or restrict the use of lived experience by PSWs within their agencies. At the same time PSWs (with few exceptions) felt frustrated by the negative messages (explicit and implicit) they recieved regarding their use of self-disclosure with service users. Some PSWs reported feeling that their potential to help service users and be efficient change agents was curtailed when they were not able to use self-disclosure (Ben-Dor *et al.*, 2024).

In another study, mental health nurses reported tensions regarding how PSWs’ lived experience should be utilized and how it impacts professional and therapeutic boundaries (Cleary *et al.*, 2018). Other studies have showen that instead of utilizing their lived-experience, a significant number of PSWs performed tasks such as administrative work, teaching skills and systems-level advocacy (Adams, 2020; Croinse *et al.*, 2016; Moran *et al.*, 2013).

Overall, despite ongoing intentional efforts to support the integration of PSWs in MH organizations, employing lived experience in mental health settings remains challenging at many levels. Power relations and organizational norms (sometimes internalized by PSWs) risk eroding the use of self-disclosure and diminishing the unique qualities that PSWs bring to MH systems. In order to support the optimal use of self-disclosure by PSWs in MH systems, I suggest addressing self-disclosure from a work environment perspective and specifically utilizing self-determination theory (Deci & Ryan, 2000).

## A way forward: addressing PSWs’ self-disclosure within a SDT perspective

Because self-disclosure lies at the crux of peer support in mental health services, self-disclosing effectively requires more than training or personal support for PSWs when facing the professional–peer paradox. PSWs need to feel comfortable when employing self-disclosure; they need to be supported by their colleagues when using self-disclosure and they need to operate within an organizational climate that explicitly endorses self-disclosure as part of a culture of recovery orientation and person-centeredness. This requires a systematic approach that is more conducive to using lived experience in services as a whole. As suggested more generally in regard to the integration of MH peer support (Adams, 2020; Byrne *et al.*, 2022; Mancini, 2018; Moran *et al.*, 2013; Tomas *et al*., 2022), effective self disclosure requires a whole-organization approach. Adopting this outlook, I suggest employing a Self-Determination Theory (SDT) perspective (Deci and Ryan, 2012) as a means to develop optimal environmental conditions that support and empower self-disclosure practices of PSWs.

SDT’s pragmatic concept of an *autonomy supportive environment* (Deci and Ryan, 2012) can be an especially useful framework to address PSWs’ self-disclosure needs. Briefly, SDT posits that individuals operate from internal motivation and thrive when their basic psychological needs for competence, autonomy and relatedness are met in *autonomy-supportive environments*. Accordingly, to enhance PSWs’ work-motivation to self-disclose and share their lived experience to benefit others’ recovery and voice service-users’ perspectives among MH staff and directors, attention should be paid to supporting competence, autonomy and relatedness – PSWs’ sense of competency in sharing one’s lived experience (e.g. feeling skilled and confident to self-disclose), their sense of autonomy to self-disclose (e.g. having a sense of freedom and choice to self-disclose) and their sense of relatedness (e.g. experiencing a positive environment that values self-disclosure) (Deci and Ryan, 2012; Gagne and Deci, 2005; Moran *et al*., 2014).

Designing autonomy-supportive environments conducive to PSWs’ self-disclosure involves developing multiple PSW roles with varying levels of self-disclosure. It also entails providing explicit support for self-disclosure and a peer nurturing culture. Such structural requirements can enable PSWs to identify roles that better fit their readiness level for self-disclosure and to more confidently pursue a career in the field of MH PSW.

For example, a recent study found that many PSWs felt empowered to share their story and utilize their lived-experience in roles that explicitly emphasize lived experience as having a peer specialty (i.e. peer expert in hospital wards, peer facilitators of a peer group intervention, etc.). Other peers felt more comfortable supporting MH service users by only sporadically sharing their lived experience aside to providing companionship. They preferred working under work roles titled ‘consumer-provider’, which involved additional tasks (e.g. assistance in daily living tasks) and did not require overtly using their lived experience unlike other peer specialist roles. Despite not using self-disclosure extensively, they still felt they could deeply connect and support service users in unique ways based on their shared lived experience (Ben-Dor *et al.*, 2024).

Another way to diversify peer roles in a single MH system can be by including peer work-roles designated to specific content-areas of self-disclosure. Examples include vocational peer support (Maru *et al.*, 2021), peer support for medical and health conditions (Druss *et al.*, 2018; Kelly *et al.*, 2014) and wellness coaching (Swarbrick *et al.*, 2016) etc. In the context of designing an autonomy supportive environment, assigning such roles within MH services can offer more opportunities for persons with lived experience that suit different needs and levels of readiness to disclose. The availability of multiple PSW roles with varying levels of saliency on self-disclosure can enhance PSWs’ sense of autonomy and comptence by offering more choice/career tracks for those contemplating engaging in peer work.

In addition, to help develop autonomy-supportive environments that enhance a positive sense of relatedness in MH services in regard to self-disclosure, organizational leaders (directors/staff heads) can role model self-disclosure by sharing relevant personal content from their own lives. Appreciation of knowledge gained from experience of colleagues and PSWs by sharing stories with staff and explicitly addressing its value can adress the challenge that arises when directors restrict PSWs from utilizing self-disclose with service users (Ben-Dor *et al.*, 2024).

Additional relatedness-building activities have been suggested, such as educating and exposing staff to the value of self-disclosure and the contribution of lived experience (e.g. Chinman *et al.*, 2017; Fortuna *et al.*, 2019; Gillard *et al.*, 2019; Mutschler *et al.*, 2022). Finally, peer supervision and peer-networking can help PSWs navigate the complexities of employing self-disclosure in statutory MH services. Dilution and co-optation processes can further be prevented when PSWs remain connected to external self-help and lived-experience groups where self-disclosure is the norm (e.g. Moran, 2017; Mutschler *et al.*, 2022, Tomas *et al*., 2022).

## Summary and conclusion

Here I described how employing SDT in regards to PSWs’ self-disclosure, specifically employing the concept of autonomy supportive environments, can guide strategies that help attenuate the professional–peer paradox. Namely, addressing PSWs’ psychological needs for competence, autonomy and relatedness in self-disclosing and designing work environments that harness multiple types of PSW roles with varying levels of self-disclosure requirements and different topic areas for self-disclosure. Multiplicity of PSW-roles can also make self-disclosure and sharing lived experience more noticeable and normalized across statutory services and systems. Additionally, promting a culture that explicilty recognizes the value of self-disclosure, such as role modelling self-disclosure by organizational leaders and expressing explicit appreciation of peer self-disclosure, can be beneficial.

Designing autonomy-supportive environments for self-disclosure may attenuate the professional-peer paradox by enabling the signature ingredient of peerness – the use of lived experience – become normative, unleashing self-disclosure’s beneficial effects within a professional setting. Overall, a culture and work-role structure that values lived-experience can help create space for PSWs’ self-disclosure at varying levels and nourish their basic psychological needs for enhancing motivation to self-disclosure. By empowering PSWs’ practice of self-disclosure we enhance their recovery-orientation and support system change.
